# Supplementary material for: Antimicrobial Effect of Copper Nanoparticles on Relevant Supragingival Oral Bacteria
Source: Microorganisms. 2024 Mar 20;12(3):624. doi: 10.3390/microorganisms12030624 (PMC10975658; doi:10.3390/microorganisms12030624)
Supplement: Supplementary file 1 [file microorganisms-12-00624-s001.zip › microorganisms-2905492-supplementary.pdf]

Table S1. Comparative Table of Antimicrobial agents used commonly over oral pathogens.

Antimicrobial agent	*S. mutans* (MIC µg/mL)	reference	*S. sanguinis* (MIC µg/mL)	reference
Sodium fluoride	625	[1]	4	[2]
Chlorhexidine	2.3	[1]	25	[3]
Penicillin	0.05	[1]	2	[4]
Chitosan	1250	[5]	1250	[5]
Chitosan NPS	625	[5]	312	[5]
Daptomycin	18.7	[3]	10.7	[3]
Cu^0 NPs	N.D. (>1000)	This job	N.D. (>1000)	Our report
Cu_2O NPs	500	This job	1000	Our report
CuO NPs	400	This job	800	Our report

1. Dong, L.; Tong, Z.; Linghu, D.; Lin, Y.; Tao, R.; Liu, J.; Tian, Y.; Ni, L. Effects of Sub-Minimum Inhibitory Concentrations of Antimicrobial Agents on Streptococcus Mutans Biofilm Formation. *Int J Antimicrob Agents* **2012**, *39*, 390–395, doi:10.1016/j.ijantimicag.2012.01.009.
2. Qian, W.; Zhang, J.; Xiao, X. [Research on Inhibition of Sodium Fluoride on Five Subgingival Bacteria in Vitro]. *Hua Xi Kou Qiang Yi Xue Za Zhi* **1998**.
3. Li, X.; Wang, Y.; Jiang, X.; Zeng, Y.; Zhao, X.; Washio, J.; Takahashi, N.; Zhang, L. Investigation of Drug Resistance of Caries-Related Streptococci to Antimicrobial Peptide GH12. *Front Cell Infect Microbiol* **2022**, *12*, doi:10.3389/FCIMB.2022.991938/FULL.
4. Doern, G. V.; Ferraro, M.J.; Brueggemann, A.B.; Ruoff, K.L. Emergence of High Rates of Antimicrobial Resistance among Viridans Group Streptococci in the United States. *Antimicrob Agents Chemother* **1996**, *40*, 891–894, doi:10.1128/AAC.40.4.891.
5. Aliasghari, A.; Khorasgani, M.R.; Vaezifar, S.; Rahimi, F.; Younesi, H.; Khoroushi, M. Evaluation of Antibacterial Efficiency of Chitosan and Chitosan Nanoparticles on Cariogenic Streptococci: An in Vitro Study. *Iran J Microbiol* **2016**, *8*, 93.
